# Social cognitive theory-based intervention to promote physical activity among prediabetic rural people: a cluster randomized controlled trial

**DOI:** 10.1186/s13063-019-3220-z

**Published:** 2019-02-04

**Authors:** Tahereh Shamizadeh, Leila Jahangiry, Parvin Sarbakhsh, Koen Ponnet

**Affiliations:** 1 Health Education and Health Promotion Department, School of Public Health, Tabriz University of Medical Sciences, Tabriz, Iran; 2 Tabriz Health Services Management Research Center, Tabriz University of Medical Sciences, Tabriz, Iran; 3 Epidemiology and Biostatistics Department, School of Public Health, Tabriz University of Medical Sciences, Tabriz, Iran; 4 Department of Communication Sciences, Imec-mict-Ghent University, Ghent, Belgium

**Keywords:** Diabetes, Prediabetes, Social cognitive theory, Physical activity

## Abstract

**Background:**

The present randomized controlled trial (RCT) evaluated the effectiveness of a theory-based physical activity (PA) intervention for rural patients with prediabetes. It was hypothesized that a PA intervention program based on the social cognitive theory (SCT) will modify fasting blood sugar (FBS) among rural people with prediabetes, which in turn will result in a decrease in diabetes incidence in the rural area.

**Methods:**

A cluster RCT on prediabetic people was conducted in Ahar, East Azerbaijan Province, Iran. A PA intervention in prediabetes was performed over 16 weeks of follow-ups in 12 villages (six per arm). Residents (*n* = 272; *n* = 136 per arm) were invited to participate in the study through rural health care centers during screening for eligibility. Participants in the intervention and control groups were informed of their prediabetic conditions and encouraged to make appropriate changes to their lifestyles to modify their prediabetes. The intervention was an educational program delivered over 16 weeks and involved behavioral change techniques. Through the education program, the intervention group received one session per week lasting about 90 min (a total of 16 sessions). The importance of risk control with PA, the duration of hill climbing, as well as exercise and safety tips were explained in a brochure that was given to the participants. Anthropometric measures, glycemic status, and PA were evaluated at the beginning of the program and after 16 weeks of follow-up.

**Results:**

The PA program showed a reduction in FBS mg/dl at 16 weeks (large-effect-size Cohen’s *d* = −0.63, *p* = 0.001) compared to the control condition. PA intervention led to a large effect size on diastolic blood pressure (BP, − 1.01) and a medium effect size for systolic BP (− 0.57), body mass index (BMI, − 0.33), and weight (− 0.35). Based on generalized linear mixed model analysis, significant reductions in FBS (mg/dl), BMI, weight, and diastolic BP were found in the intervention group compared to the control group.

**Conclusion:**

Our results support the effectiveness of an SCT-based PA intervention to reduce the risk of prediabetes developing into diabetes among rural patients with prediabetes. Findings suggest that implementation of SCT-based PA intervention for a rural population at risk of diabetes has potential benefits.

**Trial registration:**

Iranian Registry of Clinical Trials, IRCT201607198132N4. Registered on 1 September 2017. Prospectively registered.

## Background

The diabetes epidemic is a major public health concern worldwide [1]. Diabetes type 2 includes a group of metabolic disorders of which the most characterized is hyperglycemia. The prevalence of type 2 diabetes has considerably increased in recent decades, reaching 285 million cases in 2010 compared with 30 million in 1985. According to the estimates of the International Diabetes Federation (IDF), the disease will reach 438 million people in 2030 [2]. The prevalence of diabetes among the adult population is 11.4%, and with the rapid growth of the disease its consequences also increase [3].

Prediabetes, which means one’s blood sugar is above the normal range but not high enough to identify the case as diabetic, puts a person in a high-risk state for developing diabetes [4]. It has been estimated that 35% of US adults older than 20 and 50% of people older than age 65 are prediabetic. Annually, around 5–10% of prediabetic people will develop type 2 diabetes at a later age [5]. Considering the irreversible consequences of the disease and its heavy social and economic costs, early diagnosis and control of diabetes will help to prevent its most dangerous symptoms. Previous studies have shown that lifestyle interventions for patients in a prediabetic stage can reduce the risk of the disease by 60% [6].

Rural populations are more susceptible to diabetes due to characteristics such as low incomes, long distances to cities and health care centers, and limited availability of sport facilities and seasonal activity. The situation becomes more problematic when these populations have less access to health facilities and expect to have fewer medical visits; they are then more exposed to the consequences of the disease [7].

Physical activity (PA) interventions have a goal of at least 150 min/week of moderate exercise to lower the risk of diabetes [8]. A meta-analysis study showed that PA interventions decreased the risk of diabetes by 15% with 20 metabolic equivalent hours (MET-hours)/week of PA [9]. Another meta-analysis based on randomized and nonrandomized controlled trials found that PA promotion was beneficial to the prevention of prediabetes; it reduced significantly oral glucose tolerance risk ratio (26%) and fasting blood sugar (FBS) and had a favorable effect on glycated hemoglobin (HbA1C), maximum oxygen uptake (VO_2_ max), and body composition [10].

Social cognitive theory (SCT) is suitable for understanding PA health behaviors due to the interactions between individual, environment, and behavior [11]. Self-efficacy, which is one of the main constructs of the theory, means the belief a person has in his or her ability to perform a particular behavior successfully and obtain the intended results. Self-efficacy is an important prerequisite for behavior change. The other constructs of the theory are task, planning, and coping self-efficacy, goal setting, and outcome expectancy. Task self-efficacy is an individual’s confidence in his or her ability to perform certain parts of a task. Coping self-efficacy is an individual’s confidence when performing tasks under challenging conditions. Goal setting enhances self-regulation, which has an impact on self-efficacy. Outcome expectancy means beliefs related to a particular behavior that lead to specific results. The modeling of the constructs highly influences PA, planning, and compliance [12, 13]. A meta-analysis of 44 studies based on SCT showed that the models accounted for 31% of the variance in PA quality in that self-efficacy and goals were the most likely to be associated with PA. In addition, the quality of studies and the intervention strategies significantly moderated the exploratory power of SCT [13].

Few data are available on PA intervention among high-risk groups, especially rural patients with prediabetes. The present randomized controlled trial (RCT) evaluated the effectiveness of a theory-based PA intervention for rural patients with prediabetes. It was hypothesized that a PA intervention program based on SCT will modify FBS among a rural population with prediabetes, which in turn will result in a decrease in diabetes incidence in the rural area.

## Methods

### Study design

This study was an RCT on prediabetic people conducted in Ahar, East Azerbaijan Province, Iran. The study was designed to assess the effect of SCT-based PA intervention on prediabetes in rural areas of Ahar, which is surrounded by many small hills. Rural communities often lack walkable environmental features such as parks and greenways or gyms and fitness facilities for PA. The study was concluded at 4 months follow-up because the results indicated early effectiveness of PA intervention on changing prediabetes condition [14].

### Study setting and randomization

**Fig. 1:**
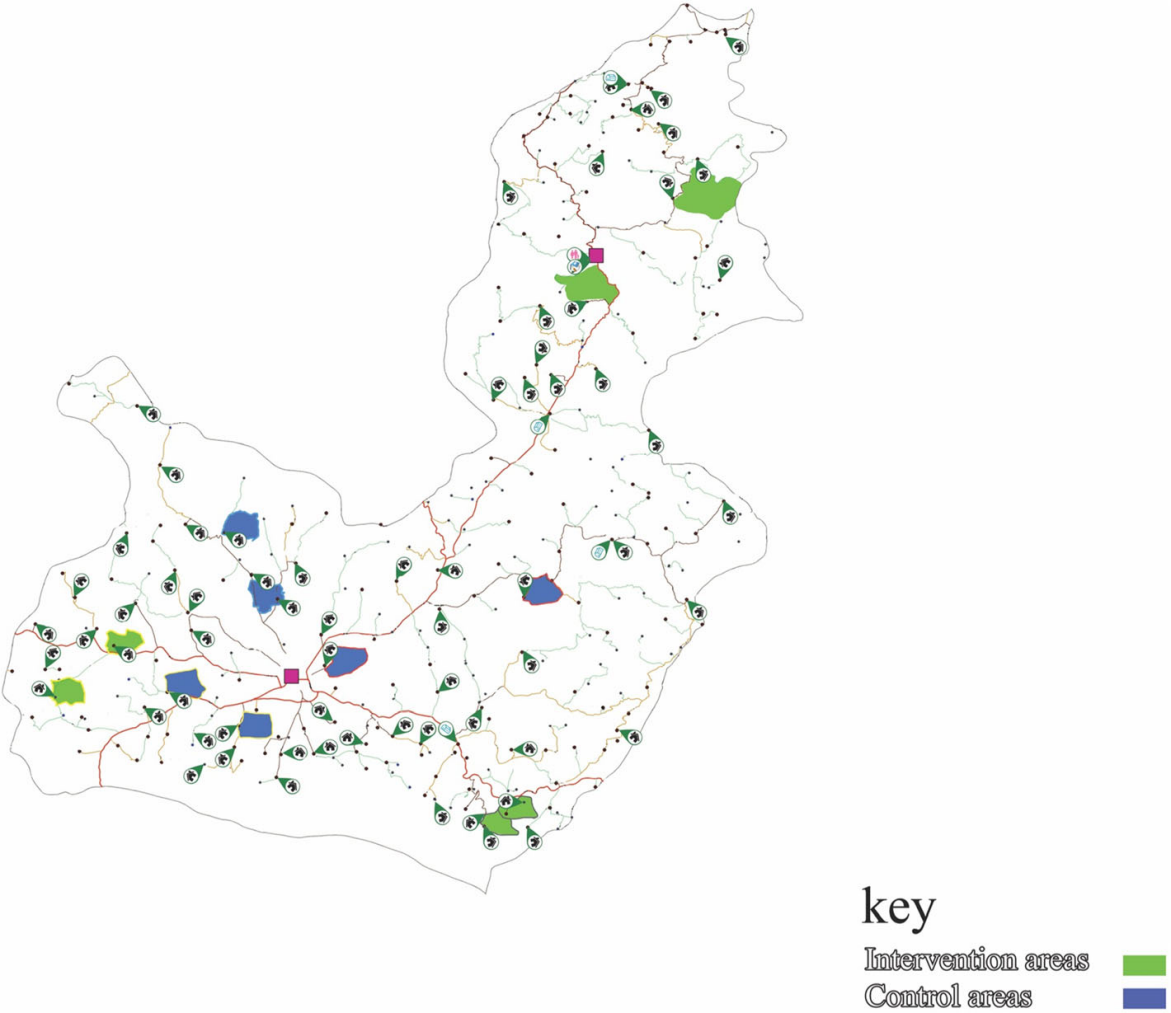
The study clusters

Ahar County is located at latitude 38.48 and longitude 47.07 in East Azerbaijan, Iran. It is situated at 1336 m above sea level with a population of 94,348, making it the third most populated county in East Azerbaijan. The study site is typical of most rural districts in East Azerbaijan. Ahar County has 13 rural health centers of which six centers were randomly selected and assigned to intervention and control groups. In this cluster RCT, the units of randomization were rural health centers with data collected from individual residents in villages. The villages covered by health centers were selected by cluster sampling and randomly allocated into intervention and control groups (in total 12 villages; six per treatment arm) by stratified block randomization (Fig. 1). The randomization process within the rural blocks was computer generated by the trial statistician. The allocation sequence was concealed from the main investigator (TSH) in sequentially numbered, opaque, sealed and stapled envelopes. The statistician was blinded at the analytic stage, and the participants were blinded to the intervention assignment; i.e., people were blinded at baseline and at 16 weeks to the allocation. Furthermore, people in the intervention group did not know that there was a control group in line with their program, and the control group also did not know there was an intervention group. The participants were told that the educational physical activity program would be conducted after 4 months.

### Recruitment and participants

**Fig. 2:**
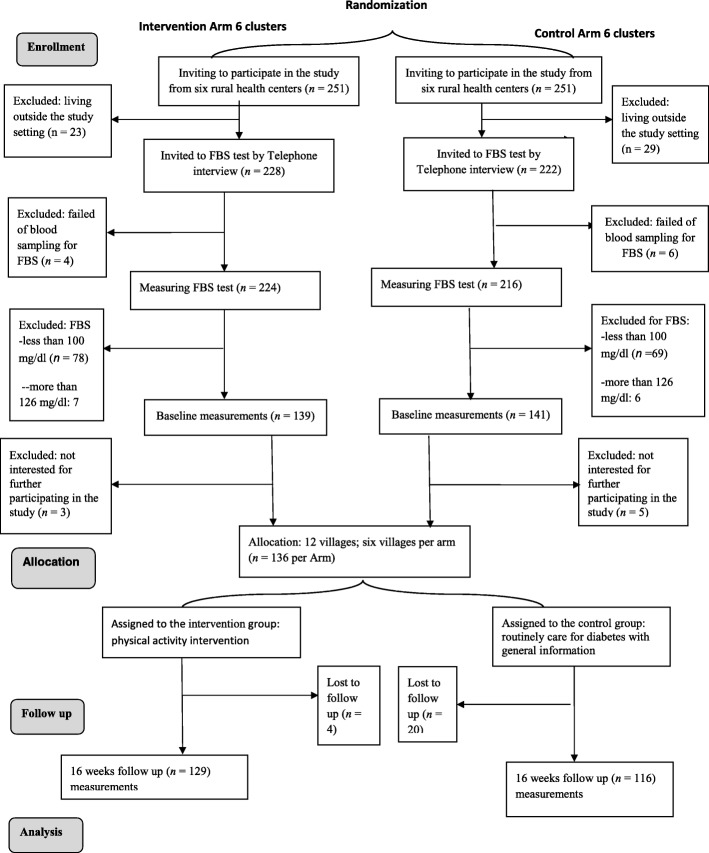
The study flowchart

The study procedure, from recruitment to data collection and follow-up assessment, is presented in Fig. 2. The referring individuals to the rural health centers were invited to participate in the study. During the first interview and eligibility screening, individuals who were interested in participating were asked to schedule a free FBS test by nursing staff in the rural health centers. A total of 136 people per arm from the selected villages who were diagnosed with prediabetes were selected to participate in the study. Participants with at least one of the following inclusion criteria were recruited to the study: a history of diabetes in family members, high blood pressure equal to or more than 140/90, obese or overweight, resident of the villages, and age 30 and over. Exclusion criteria were a disabled condition or limitation in movement and pregnancy. The study purpose was explained to the people, and their consent to participate in the study was obtained.

### Intervention

Participants in the intervention and control groups were informed of their prediabetes conditions and encouraged to make appropriate changes to their lifestyles to modify their prediabetes. The design of the intervention was guided by SCT [11] and the implementation of multiple behavioral change techniques [15] to strengthen the intervention. The intervention was an educational program delivered over 4 months and involved behavioral change techniques including the following: providing information about prediabetes, informing of the consequences of prediabetes progression to diabetes, setting graded tasks and goals for PA, and helping to control glycemic levels. The graded tasks and goals included detailed planning of frequency, intensity, and duration of PA. Prediabetes-specific education took place in rural health houses focusing on prediabetes risk assessment and targeted recommendations for safe and effective PA. Through the education program, the intervention group received one session per week that lasted about 90 min (a total of 16 sessions). The purpose of the study was explained in the first session. Participants in the intervention group were encouraged to do regular PA and meet the World Health Organization’s global recommendations. Participants in the intervention group were instructed to do at least 150 min of moderate-intensity PA per week in the morning (6:00 am to 11:59 am) or afternoon (12:00 pm to 5:59 pm) [16]. They also were encouraged to support each other to perform hill climbs that were available for people in the targeted rural community. In particular, the importance of risk control with PA, the duration of hill climbing, and exercise as well as safety tips were explained in a brochure that was given to the participants.

### Outcome measures

The primary outcome of the study was the decrease in participants’ FBS. All participants were asked to fast for 10–12 h the night before the test. Prediabetes was defined as a current fasting plasma glucose level based on FBS cut points of 100 mg/dl to 125 mg/dl [17]. Other outcomes were body mass index (BMI), weight, PA, self-efficacy, goal setting, and outcome expectancy.

Weight was measured (scale, model 8811021658; Seca, Hamburg, Germany) with the least amount of clothes and without shoes [18]. BMI was calculated as weight (kilograms) divided by the square of height (meters) [18]. Height was measured without shoes using a stadiometer (Seca, Germany) to the nearest tenth of a centimeter.

PA was measured during the last 7 days by the International Physical Activity Questionnaire (IPAQ) long form, which is a well-validated questionnaire in Iran [19]. The IPAQ long form includes 27 items in the four categories of vigorous activity, moderate activity, walking, and sitting time, which cover four domains of PA: activity at work (seven items), transportation (six items), household/gardening (six items), and leisure time activities (six items). The IPAQ data were converted to metabolic equivalent scores (MET-min/week). For estimating PA (MET-min/week) for each type of activity, the following values were used: vigorous PA = 8.0 METs, moderate PA = 4.0 METs, and walking = 3.3 METs [20].

We used standardized, structural questionnaires based on SCT to examine the related factors [21, 22]. The factors can be classified as self-efficacy, outcome expectancy, and goal setting subscales. Self-efficacy had three subscales with seven items including task, coping, and scheduling self-efficacy. *Task self-efficacy* is an individual’s confidence in performing elemental aspects of PA (i.e., following directions to complete PA). *Coping self-efficacy* is confidence when conducting PA under challenging circumstances (i.e., doing PA when one feels one has too much work to do), and *scheduling self-efficacy* is confidence in performing regular PA in spite of other time demands (i.e., doing PA when one feels one does not have time). All items began with the stem question “How confident are you that you can. ..? ” to assess the self-efficacy of the patients with five-point responses (ranging from 1 = *completely uncertain* to 5 = *completely certain*). Outcome expectancy was assessed by one question asking “To what extent do you agree to do PA at least four days of the week for 30 min a day, which is very important to you to control your prediabetes?” using a seven-point Likert scale (1 = *do not agree at all* to 7 = *completely agree*). Goal setting for PA was assessed by rating the statement “I expect to do PA most days of the week for at least 30 min per day in the next month” using a seven-point Likert scale (1 = *definitely do not* to 7 = *definitely do*).

The questions have been shown to have a good internal reliability among rural prediabetic participants (α = 0.94). The content validity was examined through consensus of teaching and research experts in health education and health promotion fields. A ten-expert panel evaluated qualitative content validity in wording, grammar, and item allocation. In the quantitative process, the content validity index (CVI) and the content validity ratio (CVR) were examined. The relevance, simplicity, and clarity of items were assessed by the CVI with four possible responses that ranged from 1 = *not relevant, not simple, and not clear* to 4 = *very relevant, very simple, and very clear* [23]. The CVI was calculated by including the proportion of items that received ratings of 3 or 4 by the experts [24]. In order to assess the essentiality of the items with the CVR, the expert panel scored each item as 1 = *essential*, 2 = *useful but not essential*, or 3 = *not essential* [24], and items with a CVR score of 0.62 or above were considered acceptable [25].

The demographic characteristics were measured by age, gender, education, income, employment, marital status, family history of diabetes, and family history of high blood pressure.

### Sample size

The effective sample size was estimated to be 136 patients per group. The study was able to detect a decrease of one standard deviation (SD; 5 mg/dl) in the FBS [26] as the most important variable of the study. A study with a power of 90% and 95% confidence and a clustering allocation design effect of 1.2 requires 136 patients per arm.

### Statistical analysis

The results were presented as mean, SD, and percentage. Normal distribution of the data was assessed using the Kolmogorov-Smirnov test and quantile-quantile plots. No values were missing at baseline, but in the follow-up, because the dropout rates were higher than 5% (6.2% in the intervention groups and 14.7% in the control group) and losses between 5 and 20% may be a source of bias [27], for the purposes of this paper the intention-to-treat (ITT) analyses were conducted using multiple imputation (MI). In this cluster randomized trial, we used a generalized linear mixed model (GMM) to compare outcome variables between groups allowing for the clustering design in our analysis and to control for confounding variables. The model was adjusted for socio-demographic characteristics (age, gender, literacy, and family history of type II diabetes and baseline measurements). The study was sufficiently powered to detect small-to-medium effects, as operationalized by Cohen’s *d* [28]. All statistical analyses were performed using STATA (version 14.0). Results were considered statistically significant at *p* < 0.05.

### Ethics

The study protocol was reviewed and approved by the Ethics Committee of Tabriz University of Medical Sciences (IR.TBZMED.REC.1395.1252) and then registered in the Iranian Registry of Clinical Trials (IRCT201607198132N4). Informed consent was obtained from all participants, and the confidentiality of the data was considered.

## Results

### Participant’s characteristics

In total, 440 individuals (from 502 invited) enrolled in the prediabetes screening program of which 168 people were excluded for not having prediabetes (*n* = 140) or not being willing to participate in the study (*n* = 28). Finally, 272 people with prediabetes agreed to be interviewed and completed the baseline measurements (see Fig. 2).

Table 1 shows that there were no significant baseline demographic differences between the intervention and control groups except for age and education. The participants in the control group (mean age 53.6 years; SD = 9.6) were significantly older than participants in the intervention group (mean age 51.3 years; SD = 11.2). Also, the control group had both more highly educated and illiterate people than the intervention group.

**Table 1:** Sample characteristics between the two groups (control and intervention)

	Intervention (*n* = 136)	Control (*n* = 136)
Age in years, mean (SD)	51.3 (11.2)	53.6 (9.4)
Gender, *n* (%)		
Women	77 (57)	85 (63)
Men	59 (43)	51 (37)
Marital status		
Married	106 (78)	111 (82)
Never married	12 (9)	3 (2)
Other	18 (13)	22 (16)
Education		
Illiterate	87 (64)	98 (72)
≤ Primary (1–6)	45 (33)	28 (21)
Secondary (7–12)	4 (3)	10 (7)
Family history, yes	35 (26)	28 (21)
Employment		
Farmer	51 (37.5)	53 (38.9)
Carpet-weavers	36 (26.4)	27 (19.8)
Animal husbandry	12 (8.8)	9 (6.6)
Worker	9 (6.6)	5 (3.6)
Not working	28 (20.5)	42 (30.8)

### The effect of PA intervention on clinical parameters and social cognitive factors

The comparison of clinical parameters and social cognitive factors between the intervention and control groups after 16 weeks PA intervention among patients with prediabetes is shown in Table 2. There were no statistically significant baseline differences in clinical outcomes except for diastolic blood pressure (BP); participants in the control group showed significantly higher diastolic BP readings (mmHg) than the intervention group.

**Table 2:** Clinical parameters and social cognitive factors between two groups of a rural population with prediabetes

Parameters	Intervention M (SD)		Control M (SD)		*p* value*	Cohen’s *d* [95% CI]
	Baseline (*n* = 136)	After (*n* = 129)	Baseline (*n* = 136)	After (*n* = 116)		
Clinical factors						
BMI, kg/m^2^	27.1 (4.8)	26.3 (4.7)	27.8 (4.3)	27.8 (4.0)	0.027	−0.33 [− 0.58 to − 0.081]
FBS, mg/dl	108.4 (6.1)	99.4 (8.1)	108 (4.8)	105.8 (8.3)	0.002	−0.63 [− 0.89 to − 0.37]
Weight, kg	68.7 (13.5)	66.9 (13.1)	71.7 (12.2)	71.2 (10.6)	0.001	−0.35 [− 0.60 to − 0.10]
Systolic BP, mmHg	129.8 (15.1)	116.5 (14.2)	132.9 (16.2)	125.2 (16.2)	0.308	−0.57 [− 0.83 to − 0.32]
Diastolic BP, mmHg	81.5 (9.4)	75.9 (8.2)	84.2 (7.2)	83.3 (6.0)	< 0.001	−1.01 [−1.28 to − 0.74]
Social cognitive factors						
Task self-efficacy	4.1 (1.68)	5.7 (0.4)	4.0 (1.2)	4.1 (1.2)	< 0.001	1.79 [1.5–2.08]
Planning self-efficacy	5.0 (1.66)	5.9 (0.9)	4.3 (1.3)	4.4 (1.3)	< 0.001	1.44 [1.16–1.72]
Coping self-efficacy	5.0 (1.5)	5.00 (1.6)	4.5 (1.3)	4.2 (1.3)	< 0.001	0.44 [0.19–0.68]
Outcome expectancy	2.1 (0.9)	3.0 (0.6)	1.9 (0.6)	2.0 (0.6)	< 0.001	1.60 [1.31–1.89]
Goal setting	2.1 (0.9)	3 (0.6)	1.9 (0.6)	1.9 (0.6)	< 0.001	1.71 [1.42–2.00]

*p value for group comparison derived from GLMM allowing for clustering design and adjusted for age, gender, literacy, and family history of type II diabetes, and baseline measurements using ITT analysis based on MI

*BMI* body mass index, *BP* blood pressure, *CI* confidence interval, *FBS* fasting blood sugar, *GLMM* generalized linear mixed model, *ITT* intention-to-treat, *M* mean, *MI* multiple imputation, *SD* standard deviation

Based on GLMM analysis, significant reductions in FBS (mg/dl), BMI, weight, and diastolic BP were shown in the intervention group compared to the control group. Systolic BP significantly decreased in the intervention and control groups after intervention, but it was not statistically significant between groups (*p* < 0.05). Also, a mixed effect model analysis allowing for the clustering was conducted without MI and without adjusting for age, education, or baseline FBS variables. The results showed no significant difference between the intervention and control groups (*p* value = 0.1). When adjusted for the mentioned confounders, a similar result was found (*p* value = 0.06).

The main PA intervention effect for FBS reached a significant level and showed a reduction in FBS mg/dl to a medium effect size (Cohen’s *d* = − 0.63, *p* = 0.001) compared to the control condition at 16 weeks. The effect sizes for BMI, weight, and systolic and diastolic BP are reported in Table 2. PA intervention led to a large effect size for diastolic BP (− 1.01) and a medium effect size for systolic BP (− 0.57), BMI (− 0.33), and weight (− 0.35). After 16 weeks intervention, there were no new diabetic patients in either the intervention or control group.

There were significant differences in SCT factors including planning and coping self-efficacy, outcome expectancy, and goal setting, but after adjusting for baseline covariates, education, and groups based on GLMM, significant improvement was detected between the two groups for all SCT factors. The effect sizes of the social cognitive factors are shown in Table 2 (*p* < 0.001).

The comparisons of PA parameters between the two groups are presented in Table 3. Significant increases in total PA, walking, and PA at work (MET-min/week) were observed at 16 weeks for the intervention group compared to the control group. The mean scores of total PA for the intervention and control groups were 9031.1 ± 4369.0 versus 7775.1 ± 4142.9, respectively. Also, the average sitting time (min/week) was significantly reduced within and between groups. All parameters were adjusted for baseline covariates, education, and groups using GLMM analysis. The effect sizes of the PA subdomains are shown in Table 3.

**Table 3:** Comparisons of physical activity between two groups of a rural population with prediabetes

Physical activity (PA) parameters	Intervention M (SD)		Control M (SD)		*p* value*	Cohen’s *d* [95% CI]
	Baseline (*n* = 136)	After (*n* = 129)	Baseline (*n* = 136)	After (*n* = 116)		
Vigorous PA (MET-min/week) median (interquartile)	3193.1 (2354.0)	2890.2 (168.3)	3410.2 (2118.8)	2811.9 (163.9)	0.739	0.029 [−0.68 to 0.127]
Intermediate PA (MET-min/week)	2886.2 (1811.4)	3119.4 (149.9)	2384.1 (1877.2)	2517.9 (161.2)	0.006	0.029 [−0.068 to 0.12]
Total PA (MET-min/week)	6776.3 (3531.1)	9031.1 (4369.0)	6643.0 (3641.9)	7775.1 (4142.9)	< 0.001	0.33 [0.23 to 0.43]
Walking (MET-min/week)	897.1 (106.1)	2366.6 (141.9)	1119.7 (119.1)	1506.8 (139.2)	< 0.001	0.51 [0.4 to 0.60]
PA at home (MET-min/week)	2695.6 (1648 .3)	2126.1 (1373.6)	2891.5 (1519.2)	2187.1 (1375.0)	< 0.001	−0.63 [−0.06 to − 0.16]
PA at work (MET-min/week), M (SD)	3903.5 (3074.9)	5891.4 (4018.1)	3553.7 (2863.0)	4489.4 (3533.4)	< 0.001	0.39 [0.29 to 0.49]
Leisure time PA (MET-min/week)	137.2 (144.3)	215. 7 (15.4)	132.0 (132.0)	226.2 (14.8)	0.629	−0.02 [−0.12 to 0.07]
Average sitting time, min/week, M (SD)	1298.2 (352.4)	620 (138.6)	1408 (358.1)	616.7 (122.9)	< 0.001	0.009 [−0.088 to 0.107]

*p value for group comparison derived from GLMM allowing for clustering design and adjusted for age, gender, literacy, and family history of type II diabetes, and baseline measurements using ITT analysis based on MI

*CI* confidence interval, *GLMM* generalized linear mixed model, *ITT* intention-to-treat, *M* mean, *MET* metabolic equivalent, *MI* multiple imputation, *SD* standard deviation

## Discussion

This study assessed the effectiveness of PA intervention based on SCT on prediabetic patients among a rural population. After 16 weeks, the intervention showed a positive impact on reducing FBS through increasing PA in the intervention group compared with the control group. Also, BMI, weight, and diastolic BP were significantly decreased in the intervention group compared to the control group. As a result, rural prediabetic patients in the intervention group participated in a prediabetes prevention program. For 16 weeks, they were made aware of diabetes risk and encouraged to do PA as a simple way to reduce the probability of developing diabetes. Our findings suggest that implementation of SCT in PA intervention has potential benefits for at-risk, unaware, and hard-to-reach people in rural areas.

The most important finding of this RCT was the reduction in FBS among rural individuals with prediabetes. The decrease was about 10 mg/dl after 4 months of PA intervention. Such decreases in FBS can reduce the economic burden of diabetes. The PA intervention program produced a relatively large effect size (Cohen’s *d* = − 0.63, *p* = 0.001) for FBS (mg/dl) levels. At least 150–175 min/week of PA reduces the risk of developing type 2 diabetes by 40–70% in people with impaired glucose tolerance [29]. Limited RCT studies have recommended that patients with prediabetes should perform approximately 150 min/week of light-to-moderate PA to lower diabetes risk [30, 31]. Chen et al. [32] reported that a 16-week empowerment program in three phases, including awareness raising, behavior building, and results checking for prediabetic patients, achieved a larger reduction in blood sugar and BMI and improved healthy lifestyle and self-efficacy significantly. Therefore, providing a theory-featured program for intervention and encouraging at-risk people to implement the recommended interventions in daily life may lead to positive outcomes. The higher effect in our study could be attributed to the SCT-based intervention for PA. As SCT explains 46% of the variance of the adults’ PA levels, and due to the finding that social cognitive variables including self–efficacy (task, planning and coping), outcome expectation, and goal setting had a strong effect on increasing PA levels [33], implementing such an intervention for at-risk prediabetic people may decrease the progression of prediabetes to diabetes. Another study systematically confirmed that action planning and providing instruction were associated with significantly higher levels of self-efficacy and physical activity. As such, it seems that self-efficacy plays a pivotal and reciprocal role in predicting PA [34].

Our trials show that provision of SCT-based information about the risk of diabetes for at-risk patients led to a large effect size on diastolic BP (− 1.01) and a medium effect size for systolic BP (− 0.57), BMI (− 0.33), and weight (− 0.35). The intervention led to moderate weight loss, which substantially reduces blood glucose level and BP. Previous evidence shows that a weight loss of 0.5–2.5 kg through lifestyle intervention, combined with an increase in PA, has beneficial effects on FBS [35, 36].

A meta-analysis of eight RCT studies showed a favorable effect on FBS (RR (risk ratio) – -0.05; 95% confidence interval, CI – 0.14 to 0.04) and HbA1C [10], but the magnitude of differences was not enough to be statistically significant (intervention group versus control group). A possible reason for this difference is related to the design and methodology of the studies. One possible explanation is related to the participants’ characteristics: in most of the studies [37–39] participants aged 40 and older were included. However, PA habits differ depending on the age of participants. More specifically, older people are more likely to remain inactive than young people [40]. Another possibility is that in the systematic review, studies used two or more interventions to reduce FBS [35, 37–39], while in our study only one intervention was included. According to Sweet and Fortier, single interventions that target physical activity or diet alone are more effective than multiple interventions [40]. The design of our study is also different from studies included in the systematic review, because cluster randomized controlled trials were excluded.

Using intervention and behavioral change techniques, such as goal setting, coping, and self-efficacy, were helpful in achieving successful results for prediabetes management [41, 42]. The findings of our study were similar to those of another study that showed significant reduction in plasma glucose among older patients with prediabetes in the intervention group during a 12-month period of synthetic intervention [43]. It could be interpreted that encouraging PA in line with current global recommendations for PA [44, 45] as well as delivering theory-based information about prediabetes control and assessing the risk of diabetes in addition to culturally tailored prevention information may motivate participants to adhere to an intervention program.

SCT is one of the most effective theories for prediction and explanation of PA behaviors [22]. The theory explains the predictors and principles of a behavior by using constructs like self-efficacy, goal setting, and outcome expectancy to guide researchers when developing educational interventions. Our study strived to include all core SCT constructs measured by the validated scales. The intervention had a significantly positive effect on all constructs of SCT in the intervention groups. Our results suggest that SCT factors are important for targeted PA behavior and prevention of type 2 diabetes. Self-efficacy is a determinant of PA behavior, so it should be emphasizedin improving PA. These findings are consistent with previous research, which has supported the relation between SCT constructs and PA [46, 47]. It seems that improving SCT factors for high-risk people at the same risk conditions for diabetes can motivate them to adopt changes in lifestyle and conduct regular PA according to the intervention program.

A previous study in Iran concluded that 8 weeks of aerobics can reduce blood glucose and cholesterol in patients with type 2 diabetes [48]. Taken together, the present study findings indicate that educating on the self-efficacy concepts (task, planning, and coping self-efficacy) and actuating people’s beliefs in the positive and beneficial change of PA can result in better blood glucose control. This cluster trial has a number of strengths. This study was conducted in a hard-to-reach and high-risk population for diabetes where diabetic patients with low income and long distances to health care centers are unable to afford health care. Another strength is that there were no new diabetic cases among prediabetic people in both groups after the 4-month intervention.

### Limitations

Insufficient previous studies on prediabetic people made it hard to compare the results of the study with others. This study was conducted on a rural population, which may limit the generalizability of the findings to urban populations. Another limitation of our study was that there were significant differences between characteristics of the control and intervention groups. This study was a cluster randomized trial from different villages.

## Conclusion

Our results support the effectiveness of SCT-based PA intervention among rural patients with prediabetes to reduce their risk of developing diabetes, through an RCT design study. Findings suggest that implementation of SCT-based PA intervention on a rural at-risk population for diabetes has the potential to benefit such a population. Further long-term research is needed to determine the maintenance of PA intervention and its impact on diabetes prevalence among rural populations.

## Abbreviations

BMI: Body mass index
CVD: Cardiovascular disease
CVI: Content validity index
CVR: Content validity ratio
FBS: Fasting blood sugar
HbA1C: Glycated hemoglobin
IDF: International Diabetes Federation
IPAQ: International Physical Activity Questionnaire
MET: Metabolic equivalent
MI: Multiple imputation
PA: Physical activity
RCT: Randomized controlled trial
SCT: Social cognitive theory
